# Hypoxia makes EZH2 inhibitor not easy—advances of crosstalk between HIF and EZH2

**DOI:** 10.1093/lifemeta/loae017

**Published:** 2024-05-06

**Authors:** Zhanya Huang, Yuanjun Tang, Jianlin Zhang, Jiaqi Huang, Rui Cheng, Yunyun Guo, Celina G. Kleer, Yuqing Wang, Lixiang Xue

**Affiliations:** 1 Cancer Center of Peking University Third Hospital, Beijing 100191, China; 2 Center of Basic Medical Research, Institute of Medical Innovation and Research, Peking University Third Hospital, Beijing 100191, China; 3 Department of Pathology, University of Michigan Medical School, Ann Arbor, MI 48109, USA

**Keywords:** EZH2, HIF-1α, hypoxia, nanoparticles, TCA cycle, drug combination

## Abstract

Histone methylation plays a crucial role in tumorigenesis. Enhancer of zeste homolog 2 (EZH2) is a histone methyltransferase that regulates chromatin structure and gene expression. EZH2 inhibitors (EZH2is) have been shown to be effective in treating hematologic malignancies, while their effectiveness in solid tumors remains limited. One of the major challenges in the treatment of solid tumors is their hypoxic tumor microenvironment. Hypoxia-inducible factor 1-alpha (HIF-1α) is a key hypoxia responder that interacts with EZH2 to promote tumor progression. Here we discuss the implications of the relationship between EZH2 and hypoxia for expanding the application of EZH2is in solid tumors.

## Introduction

Cancer is a multifaceted and heterogeneous disease that is influenced by both hereditary and environmental factors [1]. Epigenetic modifications are known to be involved in regulating gene expressions in response to various environmental cues [2]. Histone methylation modification is associated with tumor cell invasion, loss of cell differentiation, and resistance to therapeutic drugs [3]. Enhancer of zeste homolog 2 (EZH2), which is a histone H3 (trimethylation of H3 on lysine 27 (H3K27me3)) methyltransferase, is often overexpressed in a number of solid tumors, such as prostate cancer, breast cancer, colorectal cancer (CRC), melanoma, endometrial cancer, and glioblastoma (GBM) [4–6]. On one hand, EZH2 forms Polycomb repressive complex 2 (PRC2) to silence tumor suppressor genes with H3K27me3 modification which is called canonical mechanism [4, 7]. On the other hand, EZH2 may work independently of PRC2 as a co-activator to enhance tumor growth [8]. Thus, EZH2 inhibitors (EZH2is) have been pursued as cancer treatments.

Several EZH2is have been investigated as potential treatments for cancer [5], including tazemetostat, which received FDA approval in 2020 [9]. Although tazemetostat has been proven to be effective in hematologic malignancies and epithelioid sarcoma, its efficacy in treating solid tumors has been limited [10]. Treatment outcomes are often affected by the degrees of tumor infiltration [11], the tumor mutation burden [12], and the composition of the tumor microenvironment (TME) [13]. One of the main differences in the TME between hematologic malignancies and solid tumors is their vascular structures [14]. Reduced functional vasculature promotes the formation of a hypoxic TME [15]. Compared to hematologic malignancies, solid tumors exhibit denser extracellular matrix (ECM) deposition [16] and an increased secretion of substances, such as histone [17], R-2-hydroxyglutarate (R-2-HG) [18], and transforming growth factor β1 (TGF-β1) [19], which further compresses blood vessels, exacerbating the hypoxia within the tumor. Therefore, hypoxia may contribute to the restricted response to EZH2i in solid tumors compared to hematologic malignancies.

The hypoxia-inducible factor (HIF) pathway is vital in oxygen (O_2_) sensing, providing a mechanism for tumor adaptation [20]. The involvement of the hypoxia/HIF-1α pathway in both diminishing drug sensitivity [21–23] and enhancing EZH2 function [24–29] implies a pivotal role of HIF-1α in modulating the link between hypoxia and the responsiveness to EZH2is. Therefore, investigating the malignant loop between EZH2 and HIF-1α [30–32] may offer insights into prospective medication combinations in solid tumors. Additionally, exploring strategies to alleviate the hypoxia within the TME may enhance the applicability of EZH2is.

### 1. EZH2 modulates the hypoxia/HIF-1α pathway

#### 1.1 Direct regulation

**Figure 1:**
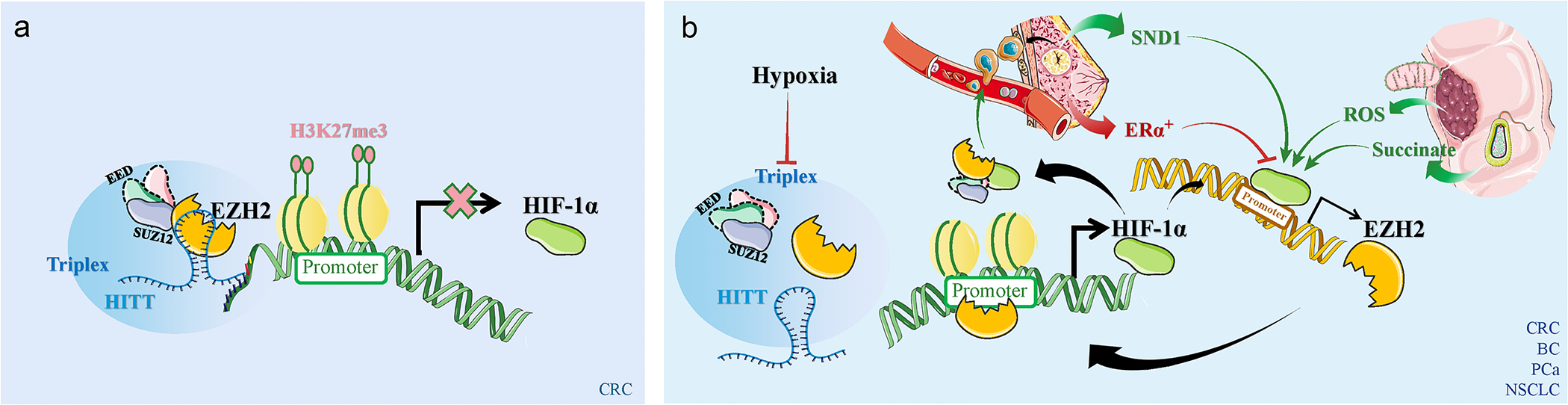
Schematic of EZH2 and HIF-1α interplay in various cancers under different oxygen conditions. (a) Under normoxia condition, EZH2 inhibits HIF-1α in CRC. (b) Under hypoxia conditions, reciprocal loop formation between EZH2 and HIF-1α in CRC, breast cancer (BC), PCa, and NSCLC. Thin red arrows denote inhibition, and thin green arrows indicate promotion. Thick arrows signify tumor-suppressive (red) or tumor-promoting (green) indicators.

Hypoxic circumstances switch EZH2 from a suppressor to an activator of HIF-1α in CRC (Fig. 1). In a normoxic environment, EZH2 is guided to the promoter region of *HIF-1α* by the long non-coding RNA (lncRNA) *HITT* (HIF-1α inhibitor at translation levels) and suppresses its transcription by forming an RNA/PRC2/DNA triplex, while EZH2 is untethered due to *HITT* degradation under hypoxia, which hinders the repressive action of EZH2 on HIF-1α transcription in CRC cells [33]. However, in gastric cancer cells, EZH2 continues to bind directly to the *HIF-1α* promoter even in the presence of hypoxia [34]. These findings suggest an environment-dependent regulation role of EZH2 on HIF-1α. In addition, in lung cancer cells, inhibition of either EZH2 or HIF-1α could enhance the other’s function [35], which suggested that the prognosis for tumors might be enhanced by simultaneously inhibiting EZH2 and HIF-1α.

#### 1.2 Indirect regulation

EZH2, always functioning within the nucleus, has been reported to indirectly enhance HIF-1α levels by suppressing the eleven-nineteen lysine-rich leukemia (ELL)-associated factor 2 (EAF2)/von Hippel-Lindau tumor suppressor protein (pVHL) axis to exert immune evasion [36, 37]. In GBM and non-small cell lung cancer (NSCLC), the inhibition of EAF2 by EZH2 leads to a reduction in pVHL, thus augmenting the protein level and activity of HIF-1α by preventing the ubiquitin-dependent degradation process. The heightened activity of HIF-1α improves the adaptability of tumors against hypoxic conditions and stimulates regulatory T (Treg) cell development via TGF-β overexpression. The presence of Treg cells in the TME diminishes the cytotoxic function of CD8^+^ T cells, thereby facilitating tumor cells to evade immune surveillance. Meanwhile, it has been discovered in NSCLC that EZH2 can also promote tumor immune evasion by enhancing HIF-1α induced programmed death ligand 1 (PD-L1) expression [38]. In light of these findings, it is worth further investigating whether EZH2is should be used in combination with immunotherapy to improve the poor prognosis induced by hypoxia.

Furthermore, two upstream regulators of this pathway, forkhead box C1 (FOXC1) and LINC00301, have been identified. FOXC1 stimulates the transcription of LINC00301, allowing the direct binding between LINC00301 and EZH2. This interaction mediates the recruitment of EZH2 to the *EAF2* promoter region, leading to the inhibition of the EAF2/pVHL pathway and the stabilization of HIF-1α in the nucleus. Moreover, elevated LINC00301 in the cytoplasm functions as a competitive endogenous RNA (ceRNA) for miR-1276, preventing the posttranscriptional inhibition of HIF-1α. Therefore, FOXC1/LINC00301 regulates HIF-1α through nuclear and cytoplasmic pathways. Notably, the nuclear levels of LINC00301 are much higher than their cytoplasmic levels, suggesting a predominant role of EZH2 in FOXC1/LINC00301-mediated upregulation of HIF-1α [37].

In addition, EZH2 promotes the accumulation of HIF-1α by inhibiting its regulator prolyl-hydroxylase (PHD3) in lung adenocarcinoma (LUAD). Specifically, the expression of HIF-1α is typically regulated by PHD3, a catalyst that prompts HIF-1α degradation in the presence of α-ketoglutarate (α-KG). However, α-KG reduction stabilizes HIF-1α in the first two hours of hypoxia. As the hypoxic condition persists for 5 days, EZH2 exerts its function of inhibiting PHD3 and disrupts the usual control mechanism which initiates a reciprocal loop with an lncRNA named HIFAL, a HIF-1α anti-sense lncRNA, resulting in amplified HIF-1α levels. Thus, EZH2 indirectly promotes the buildup of HIF-1α, which causes tumor cells to dedifferentiate by blocking PHD3 [39].

### 2. The function of EZH2 varies from normoxia to hypoxia

As previously stated, there is an environment-dependent regulation role of EZH2 on HIF-1α, with hypoxia being one of the influencing factors. For example, within colorectal TME, EZH2 suppresses HIF-1α in physiologically normal settings (normoxia) but promotes HIF-1α’s activity in hypoxic situations [33, 34, 36, 37]. Understanding how EZH2’s function shifts within the hypoxic TME is critical for gaining a deeper understanding of the complex interplay between the two proteins.

#### 2.1 Hypoxia augments the metastasis-promoting role of EZH2

Hypoxia accelerates breast cancer metastasis via EZH2. The circadian clock gene Period2 (*PER2*) is known to recruit EZH2 to bind the octamer transcription factor 1 (OCT1). This facilitates the methylation-dependent silencing of epithelial-mesenchymal transition (EMT) genes such as snail family zinc finger 1 (*SNAIL*), snail family zinc finger 2 (*SLUG*), and twist family bHLH transcription factor 1 (*TWIST*). Under hypoxic conditions, however, PER2 levels decrease due to degradation, disrupting the inhibitory function of EZH2 on EMT genes and increasing tumor invasiveness [24].

#### 2.2 Hypoxia boosts the EZH2-mediated promotion of tumor drug resistance

EZH2 facilitates tumor drug resistance during hypoxia with the activation of growth factor pathways [25] and the dedifferentiation of tumor cells [26]. It has been reported that hypoxia in pancreatic cancer triggers the activation of pancreatic stellate cells (PSCs), leading to the secretion of exosomes enriched in lncRNA urothelial carcinoma-associated 1 (*UCA1*). *UCA1* is subsequently transported to pancreatic cancer cells by these exosomes. Once entering the cells, *UCA1* recruits EZH2 to silence the transcription of the suppressor of the cytokine signaling 3 (*SOCS3*) gene [25]. This indirectly activates the Janus kinase 2 (JAK2)/signal transducer and activator of transcription 3 (STAT3) signaling pathway [27], allowing pancreatic cancer cells to become more treatment-resistant.

Regarding the dedifferentiation of tumor cells during drug resistance, it was reported that when treated with nutrient restriction and hypoxia, ^V600E^*BRAF* mutant melanoma cells were shown to have tumor vascular disruption. Tumor cells within the hypoxic regions show lower levels of glutamine, disrupting the tricarboxylic acid (TCA) cycle. Consequently, the level of α-KG decreases, which may block the activity of demethylases (Jumonji-domain-containing (JmjC) histone demethylases (jHDMs)). The action of JmjC proteins increases the overall level of histone methylation, with a particularly large increase in EZH2-mediated methylation (H3K27me3). This increased level of H3K27me3 in melanoma cells triggers their dedifferentiation and confers resistance to BRAF inhibitors [26].

#### 2.3 Prognostic significance and immune implications of EZH2 in hypoxic tumor models

According to a bioinformatics study, EZH2 has been found to have many oncogenic functions and is a prognostic marker for different types of solid tumors. This indicates that by measuring the level of EZH2 expression, we can forecast the prognosis of tumors and stratify patients to support clinical decision-making. In addition to identifying differential genes through public database scanning, other studies focus on malignant features of tumors for prognostic model development or drug target selection. For instance, five characteristic genes (cyclin-dependent kinase 1 (*CDK1*), *EZH2*, cyclin B1 (*CCNB1*), cyclin A2 (*CCNA2*), and Aurora kinase A (*AURKA*) were evaluated according to the stemness traits of tumor cells in triple-negative breast cancer (TNBC) and may serve as prognostic indicators or therapeutic targets [29]. The receiver operating characteristic (ROC) and correlation analyses suggested that these five genes among which EZH2 exhibits a substantial positive correlation with hypoxia signatures, can predict low O_2_ levels in TNBC. Therefore, assessing EZH2 levels in TNBC patients can predict the degree of stemness and hypoxia in TNBC cells in addition to evaluating the prognosis of the patients. Moreover, a positive correlation between hypoxia and EZH2 in liver cancer was also found when a predictive model was constructed utilizing hypoxia-related genes (HRGs) [28]. Specifically, four HRGs (decorin (*DCN*), DNA damage inducible transcript 4 (*DDIT4*), protein kinase C alpha (*PRKCA*), and N-myc downstream regulates gene-1 (*NDRG1*)) were included in the hypoxic risk model for liver cancer that the researchers created. Immune cell infiltration scores were lower in patients with greater hypoxia risk model scores. This phenomenon can be partly attributed to the increased expression of EZH2. As revealed by the single-cell sequencing database Human Protein Atlas (HPA) and correlation analysis, EZH2 is favorably connected with hypoxia risk scores and negatively correlated with immune infiltration scores. Therefore, assessing EZH2 expression levels can help further differentiate the malignancy of tumor cells under hypoxic conditions from the perspective of immune infiltration. However, these discussions still require further experimental validation.

Additionally, the intermediary metabolites between EZH2 and hypoxia were discovered to contain potential targets for posterior fossa A (PFA) ependymomas [40]. Hypoxia improves S-adenosyl-methionine (SAM) sequestration and raises α-KG and acetyl-CoA levels, and the altered metabolite landscape consequently leads to a decrease in H3K27me3 level and an increase in acetylation of lysine 27 on histone H3 (H3K27ac) level. Thus, analyzing these metabolites may aid in developing more effective treatment strategies and biomarkers for children with PFA ependymoma.

### 3. Mechanisms of how HIF-1α modulates EZH2

As previously mentioned, hypoxia amplifies or reduces the canonical role of EZH2 (H3K27me3) by regulating α-KG levels [26, 40]. However, HIF-1α could directly modify the function of EZH2, either by enhancing its activity or by transitioning its role from canonical to non-canonical role [41–45].

#### 3.1 Direct regulation

HIF-1α directly regulates EZH2 through direct recruitment and transcriptional upregulation (Fig. 1).

Firstly, HIF-1α enhances *EZH2* transcription by binding to a hypoxia response element (HRE: NCGTG) near the *EZH2* promoter region under hypoxia leading to increased *EZH2* expression [41]. Several variables within the hypoxic TME, including SND1 (staphylococcal nuclease domain-containing protein 1), estrogen receptor α (ERα)/forkhead box K2 (FOXK2), succinate, and reactive oxygen species (ROS), can boost or decrease this direct regulation. HIF-1α inhibits Rad51 and induces DNA breaks in early-stage breast cancer cells, notably in breast tumor-initiating cells (BTICs) by targeting EZH2. This may subsequently activate the RAF1 (C-RAF)/extracellular signal-regulated kinase (ERK)/Wnt pathway, promoting the proliferation of BTICs [41]. In addition, SND1, a known pro-oncogenic protein, augments the binding of HIF-1α to EZH2, contributing to the development of breast cancer induced by the polyomavirus middle T antigen (PyMT) [42]. Consequently, overexpression of *SND1* further enhances the activity of the HIF-1α/EZH2 axis in advancing BTICs. Moreover, HIF-1β, the functional co-subunit for HIF-1α, is suppressed by functioning ERα specifically transactivating FOXK2 which reduces the HIF-1/EZH2 pathway [31]. Therefore, the HIF-1/EZH2 signaling can be sustained inside extremely aggressive breast cancer cells devoid of the estrogen receptor (ER^−^), leading to the proliferation, invasion, migration, and angiogenesis of breast cancer cells [46]. This highlights the significance of the HIF-1/EZH2 signaling in the poor prognosis of ERα^−^ breast cancer patients and the potential for drug combination to intervene in the ERα-FOXK2-HIF1β/EZH2 axis. The upregulation of EZH2 by HIF-1α is also observed in prostate cancer (PCa) cells while exposed to hypoxia. Elevated EZH2 expression led to the silencing of a type II TGF-β receptor (TGFBR2), a transmembrane serine-threonine kinase, and upregulating of genes associated with EMT, which enhances the invasion of PCa cells [47].

The mechanism described above is intimately connected to carbohydrate metabolism. On the one side, increased EZH2 levels in NSCLC produced by HIF-1a can suppress F-box/LRR-repeat protein 7 (FBXL7), an E3 ubiquitin ligase responsible for 6-phosphofructo-2-kinase/fructose-2,6-biphosphatase 4 (PFKFB4) degradation. Increased PFKFB4 levels typically promote glycolysis within TME. As a result, HIF-1α/EZH2 signaling facilitates glycolysis in NSCLC cells by inhibiting FBXL7 and stabilizing PFKFB4 [48]. On the other side, various TCA cycle intermediates, such as succinate [49] and ROS [50], can enhance the HIF-1α/EZH2 axis. Firstly, for succinate, this study incorporated the microbiota into the network of interactions between immune cells and tumor cells and discovered that *Fusobacterium nucleatum* could promote immune evasion by upregulating the succinate/HIF-1α/EZH2 axis within CRC cells, despite the unclarified mechanism [49]. Generally, the succinate buildup stabilizes HIF-1α by interfering with PHD function [50]. An increased expression level of EZH2 inhibits the cyclic guanosine monophosphate-adenosine monophosphate (GMP-AMP) synthase (cGAS) activity, dampening the cGAS-stimulator of interferon genes STING- interferon-β (IFNβ) pathway and resulting in lower immunotherapy sensitivity among CRC patients [49]. Therefore, the combination use of EZH2i with metronidazole or fecal microbiota transplantation (FMT) could be taken into consideration. As for ROS, increased ROS release from mitochondria was observed upon exposure of CRC cells to hypoxia. This increased ROS activated the HIF-1α/EZH2 pathway by enhancing the HIF-1α protein stability and the interaction between HIF-1α and the *EZH2* promoter region, resulting in the upregulation of EZH2. Interestingly, the levels of ROS were elevated in most solid tumor types, suggesting that the reinforcement of the HIF-1α/EZH2 pathway may be prevalent [51]. In addition, glucose deprivation can lead to pseudohypoxia, which promotes EZH2 to upregulate HIF-1α in LUAD [39].

Secondly, when exposed to hypoxia, elevated HIF-1α levels induce the recruitment of EZH2 and the suppressor of zeste 12 (SUZ12) to silence methylation-mediated cell adhesion-related protein desmoglein2 (DSG2). As a consequence, primary tumor cells located within hypoxic regions acquire the capability to enter the circulation. These tumor cells may migrate to normoxic regions in the bloodstream where DSG2 expression is restored, allowing them to settle in distant organs [52]. This dynamic process underlines the role of HIF-1α in regulating EZH2-mediated epigenetic modifications that contribute to tumor cell dissemination and metastasis.

#### 3.2 Indirect regulation

##### 3.2.1 Mediated by transcriptional factors (TFs)

HIF-1α was found to promote EZH2 expression by upregulating several TFs, including TWIST and metastasis-associated protein 2 (MTA2) under hypoxic TME. For instance, HIF-1α may upregulate the expression of TWIST by interacting with really interesting new gene 1B (Ring1B) and EZH2 in pancreatic ductal adenocarcinoma (PDAC). As a result, E-cadherin and p16 are deacetylated and their transcriptions are repressed, leading to more proliferative and invasive cancer cells [43].

HIF-1α upregulates *MTA2* transcription in PCa by binding to the HRE of *MTA2*. Increased MTA2 has two primary functions: firstly, it forms the nucleosome remodeling and histone deacetylase (NuRD) complex with histone deacetylase 1 (HDAC1) to inhibit acetylation-dependent degradation of HIF-1α [53]. Secondly, MTA2 can recruit EZH2 to methylate and silence the inhibitors of the mechanistic target of rapamycin (serine/threonine kinase) (mTOR) pathway, including phosphatase and tensin homolog (PTEN), tuberous sclerosis 2 (TSC2), Ras homolog gene family member A (RHOA), DEP domain-containing mTOR-interacting protein (DEPTOR), FK506 (tacrolimus)-binding protein 11 (FKBP11), G-protein signaling regulator 16 (RGS16), and glycoprotein 1 (GP1) [54]. Given that the mTOR pathway is a classical upstream mechanism for HIF-1α [55], EZH2 may indirectly enhance the transcription of *HIF-1α* by upregulating the mTOR pathway in a PRC2-dependent manner. Overall, these research findings suggest that MTA2 may contribute to the reciprocal loop between HIF-1α and EZH2 in PCa.

##### 3.2.2 Epigenetic modification

HIF-1α can either promote or recruit EZH2 through non-coding RNAs (ncRNAs), chromatin scaffolds, and histone erasers.

Some ncRNAs, such as FOXD2-AS1 and miR-101, have been reported to be involved in the HIF-1α/EZH2 signaling pathway. It was suggested that HIF-1α promotes the transcription of the lncRNA FOXD2-AS1 through direct binding to the promoter of *FOXD2-AS1* [56]. The EZH2 protein is recruited to p21 by elevated FOXD2-AS1, causing the methylation and subsequent silencing of p21. This process is dependent on the PRC2 complex, potentially promoting the proliferation of osteosarcoma cells [56]. HIF-1α could also enhance the transcription of *EZH2* by downregulating miR-101, a well-known EZH2 suppressor. This process could be influenced by the expression level of androgen receptor (AR) and vascular endothelial growth factor (VEGF)/VEGF receptor 2 (VEGFR-2) pathway to affect prognosis [32, 57]. Androgen stimulation raises miR-101 levels to suppress EZH2 expression, improving PCa prognosis in AR-positive LNCaP cells [32]. Furthermore, the elevation of HIF-1α/HIF-1β not only reduces miR-101 expression but also indirectly stimulates EZH2 transcription in PCa cells. AR-negative PC-3 cells and AR-positive but prostate-specific antigen (PSA)-negative DU145 cells were utilized in this experiment. These findings may assist in understanding why PCa patients respond differently to therapy [32, 57]. Furthermore, the HIF-1α/miR-101/EZH2 interactions in LUAD are greatly influenced by the VEGF/VEGFR-2 pathway. HIF-1α can upregulate EZH2 expression in LUAD cells expressing VEGFR-2, which is not dependent on hypoxia. By decreasing miR-101, the VEGF/VEGFR-2 pathway indirectly stimulates EZH2 production. This indicates that the HIF-1α/miR-101/EZH2 network is active even under normal O_2_ circumstances in lung cancer cells (HCC4006, HCC461, and HCC1171) that are subjected to increased VEGF stimulation and high levels of VEGFR-2 expression. Consequently, *EZH2* overexpression in these circumstances may lead to resistance to combination therapies that incorporate cisplatin, carboplatin, and AZD2171 treatments in patients with LUAD [57].

In clear cell renal cell carcinoma (ccRCC) and TNBC, hypoxia and HIF-1α play a pivotal role in amplifying the metastatic potential of tumor cells through non-canonical functions and enhanced EZH2 phosphorylation. The involvement of a chromatin scaffold protein, Zinc finger MYND (Myeloid, Nervy and DEAF-1)-type containing 8 (ZMYND8), further enhances this process. Specifically, HIF-1α selectively hinders the production of PRC2 subunits SUZ12 and embryonic ectoderm development (EED), promoting the formation of the EZH2/FoxM1 complex. This interaction transforms the canonical function (PRC2-dependent) of EZH2 into a non-canonical (PRC2-independent) way [44]. ZMYND8 overexpression in ccRCC reinforces this shift in functionality, acting as a scaffold protein to facilitate the EZH2/FoxM1 complex formation [45]. Moreover, EZH2 phosphorylation at T487 intensifies this effect of ZMYND8 [45]. Consequently, under hypoxia or in the presence of HIF-1α, EZH2 transitions from repressing matrix metalloproteinases (MMPs) to activating MMPs in an EZH2/FoxM1-dependent pathway [44, 45]. These studies show a novel effect of hypoxia and HIF-1a in enhancing EZH2 phosphorylation and non-canonical functions. The role that hypoxia plays on other EZH2 phosphorylation and PRC2 independent functions of EZH2, such as EZH2 T367 phosphorylation [58] and EZH2-mediated nuclear factor κB (NF-κB) activation [59], warrants further investigation.

Lysine demethylase 8 (KDM8)/JumonjiC (JmjC) domain-containing protein 5 (JMJD5) has been implicated in two distinct functions in castration-resistant PCa (CRPC) with EZH2 and HIF-1α. CRPC is a PCa subtype with a poor prognosis. It exhibits resistance to androgen deprivation therapy (ADT) but remains AR-dependent. This dependence is frequently linked to AR reactivation via a variety of mechanisms. KDM8/JMJD5, which is increased in CRPC, activates AR in the absence of androgens and acts as a co-activator, upregulating androgen-responsive genes such as AAA nuclear coregulator cancer associated protein (*ANCCA*)/ATPase family AAA domain-containing protein 2 (*ATAD2*) and *EZH2*. Consequently, it sustains the viability of AR-dependent CRPC cells [60]. In addition, KDM8/JMJD5 has been observed to interact with pyruvate kinase M2 (PKM2) to promote PKM2 translocation into the nucleus to form the KDM8/PKM2 complex. The KDM8/AR/EZH2 axis interacts with the transcription factor HIF-1α, leading to increased expression of glycolytic genes. The ensuing metabolic adaption helps PCa cells survive. Given these results, the KDM8/AR/EZH2 axis may play a role in several malignant alterations found in CRPC, including metabolic reprogramming, increased expression of neuroendocrine factors, and induction of pro-tumorigenic factors [60]. Interestingly, in the physiological cycle of the ovary, AR-mediated upregulation of HIF-1α can suppress the canonical function of EZH2 by promoting the expression of KDM6B/JMJD3 [61], highlighting the context-dependent nature of HIF-1α/EZH2 interaction.

### 4. Hints for clinical application

#### 4.1 Drug combination strategy

The malignant feedback loop formed by the proteins EZH2 and HIF-1α often leads to unfavorable prognoses in a variety of cancers. Because the EZH2/HIF-1α feedback loop is particularly prominent in individuals with very aggressive and difficult-to-treat malignancies, such as CRPC patients and ERα-negative breast cancer patients, it is believed that the EZH2/HIF-1α interaction contributes to the reduced sensitivity to drug treatments. Therefore, exploring a combined drug strategy of inhibiting EZH2 and HIF-1α together could potentially improve the effectiveness of cancer treatment.

##### 4.1.1 Interference of EZH2

EZH2is are classified into three main categories, including: (1) those inhibiting the EZH2 enzyme activity, (2) those destabilizing the PRC2 complex, and (3) those degrading the EZH2 protein itself.

Conventional EZH2is inhibit the enzymatic activity of EZH2 by blocking its methionine cycle. These inhibitors could be SAM-based (e.g., EPZ005687, UNC1999, GSK126 (GSK2816126), EI1, CPI-1205, tazemetostat (E7438/EPZ6438), and MC3629) or S-adenosyl-L-homocysteine (SAH)-based (e.g., 3-deazaneplanocin A (DZNep)) [62]. Tazemetostat has been approved by the FDA for treating several types of lymphoma [63]. Inhibitors targeting the PRC2 complex, such as A-395 [64], disrupt the interactions between EZH2 and other PRC2 subunits (SUZ12 and EED), destabilizing the PRC2 complex and reducing its catalytic activity. Besides, chemicals that destabilize EZH2 and inhibit its non-canonical activities have been developed as a result of a better knowledge of its PRC2-independent role [65]. For example, MS177 [66] and MS1943 [67] have been developed utilizing the heterobifunctional PROTAC (proteolysis-targeting chimera) technology. These compounds act as molecular bridges connecting EZH2 to ubiquitin ligases (Cereblon or VHL), resulting in the ubiquitination of EZH2 and its subsequent degradation within the proteasome.

##### 4.1.2 Interference of HIF-1α

Researchers are actively exploring molecules that could modulate HIF-1α functionality. These compounds can be grouped into three major categories: non-specific upstream inhibitors, direct inhibitors of HIF-1α, and downstream inhibitors.

Type I compounds regulate HIF-1α through its non-specific upstream signaling, leading to a decrease in its expression or activity. These include inhibitors of the phosphatidylinositol 3-kinase (PI3K)/protein kinase B (AKT) pathway (such as LY294002 and wortmannin) and inhibitors that act on the mTOR pathway (such as temsirolimus, everolimus, and sirolimus) [68]. Type II compounds inhibit the transcription or translation of HIF-1α. For instance, EZN-2698 and aminoflavone inhibit the transcription of HIF-1α [69, 70], while cardiac glycosides, 2-methoxyestradiol (2-ME2), and camptothecins prevent the translation of HIF-1α protein [71–73]. Type III inhibitors act downstream of HIF-1α by disrupting the interaction with HIF-1β to suppress the activity of HIF-1α. These include Hsp90 inhibitors, HDAC inhibitors (HDACis), and acriflavin [74].

##### 4.1.3 Drug combinations

Some chemotherapy regimens, involving the inhibition of both HIF-1α and EZH2, have been developed. For example, HDACi as a type III inhibitor was combined with EZH2i to improve prognosis in cancer patients with GBM and breast cancer [75, 76]. But rather than emphasizing the HDACi’s inhibitory effect on HIF-1α, the main mechanism of this combination was the increased production of tumor suppressors such as interferon-γ inducible protein 16 (IFI16) via up-regulating H3K27ac and down-regulating H3K27me3 levels [76]. Besides, recent studies found dietary and herbal compounds, such as apigenin [34] and a synthetic derivative of curcumin called CDF (a novel curcumin-derived synthetic analogue) [77], could simultaneously inhibit HIF-1α and EZH2, reducing cancer invasiveness and improving tumor prognosis. Notably, a small molecule compound, DYB-03, was designed to dually target EZH2 and HIF-1α [35]. DYB-03 showed better chemotherapeutic effects compared to either 2-ME2 (HIF-1α inhibitor (HIF-1αi)) or GSK126 (EZH2i) in NSCLC.

These studies provide the feasibility of combining EZH2i and HIF-1αi and offer valuable insights. However, in specific tumors like NSCLC, the regulatory role of EZH2 on HIF-1α mainly manifests as inhibition of T cells [37, 38]. In such cases, the combination of EZH2i and hypoxia-related chimeric antigen receptor (CAR)-T technology could be an alternative treatment. For instance, CAR-T cells engineered with an oxygen-dependent degradation domain (ODD) and an HRE exhibit enhancement of the efficacy of immunotherapy in hypoxic conditions which are often governed by HIF-1α. It is a promising technique for treating the TME [78]. Notably, several challenges, such as determining which types of tumors respond to this combination, as well as defining the optimal timing and duration of treatment, need to be further solved. These aspects necessitate further validation.

#### 4.2 Application of nanoparticles (NPs) in hypoxia relieving

Hypoxia not only promotes EZH2 expression via HIF-1α but also amplifies its pro-tumorigenic properties. For instance, ROS and SND1, which are produced in the hypoxic TME, can further upregulate EZH2 [42, 49]. Therefore, developing alternative therapeutic strategies that relieve hypoxic TME may be an effective way to improve the outcome of EZH2 inhibition in cancer treatment.

**Figure 2:**
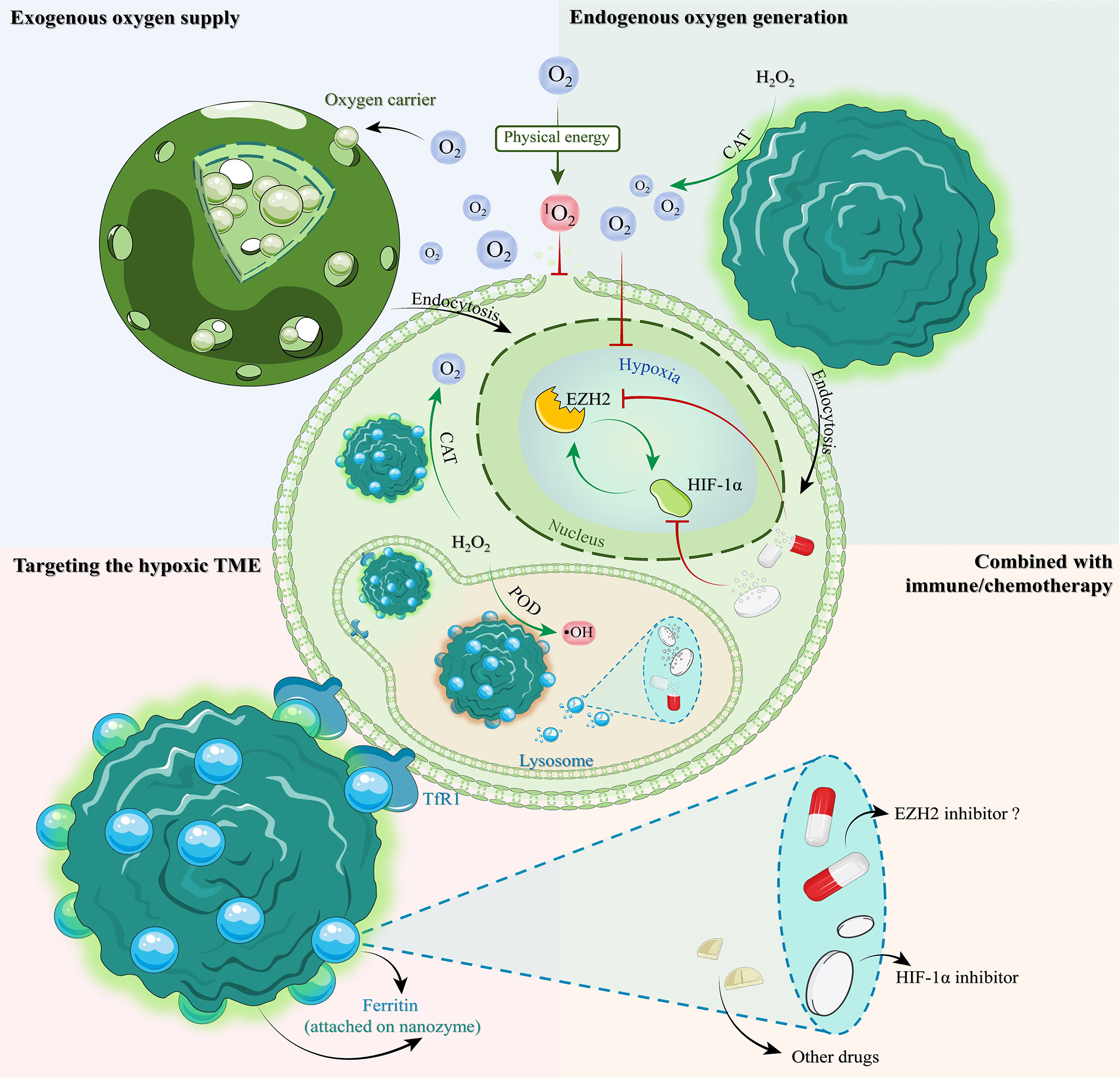
Nanotechnology strategies for hypoxia alleviation. This figure illustrates three nanotechnology strategies (exogenous release of oxygen, endogenous catalytic oxygen production, and targeting the hypoxic TME) combating tumor hypoxia. These nanotechnologies, symbolized by arrows (red for inhibition, green for promotion), can also encapsulate drugs (combined with immune/chemotherapy), suppressing the malignant loop between HIF-1α and EZH2. Physical energy sources (radiotherapy, phototherapy, ultrasound) activate O_2_ into ^1^O_2_ to disrupt the cell membrane. NanoparticlesN inside tumor cells exhibit POD-mimic activity (POD) in lysosomes for their acidic environment (shown in orange) and CAT-mimic activity (CAT) upon lysosomal escape.

NPs are known for their good biocompatibility, bioavailability, and favorable surface area-to-volume ratio, properties making them valuable for enhancing drug delivery and reducing drug toxicity [79]. With technological advancements, the NPs’ potential to improve the TME and solve problems including acidity [80], vascular abnormalities [81], and hypoxia [82] has also been investigated. The three major methods for modifying the hypoxic environment by NPs are exogenous oxygen supply, endogenous catalytic oxygen generation, and hypoxic TME targeting delivery (Fig. 2).

Exogenous oxygen-supplying NPs come in various forms. Since perfluorocarbon (PFC) could serve as an O_2_ reservoir and release O_2_ gradually, many PFC-based NPs are designed to relieve the hypoxia in solid tumors. TaOx@PFC–PEG, for example, utilizes PFC to supply O_2_ and TaOx to concentrate radiation energy to enhance radiotherapy [83]. Similarly, in the scaffolding system of perfluorooctane emulsion (O_2_ carrier)-loaded hollow microparticles (PFO-HPs), PFO supplies enough O_2_ to cells attached to the HPs to prevent them from hypoxia-induced necrosis [82]. To manage O_2_ release, a photothermally controlled “oxygen bomb” PSPP-Au980-D was recently developed. It employs a 980 nm laser to trigger O_2_ release and a 680 nm laser to convert O_2_ to singlet oxygen (^1^O_2_) for photodynamic therapy (PDT) [84].

Exogenous oxygen-supplying NPs have limited capacity, so novel NPs, called nanozymes, were designed to catalytically generate O_2_ in the hypoxic TME. Such endogenous catalytic O_2_-generating nanozymes include MnO_2_-based NPs exhibiting catalase (CAT) activity, such as MnO_2_ NPs [85], iPS-MnO_2_@Ce6 [86], and H-MnO_2_-PEG/C&D [87]. NPs with electron-hole separation ability or light absorption ability can further activate O_2_ to cytotoxic ^1^O_2_ under ultrasound or light irradiation [88]. These CAT-like nanozymes do, however, depend on hydrogen peroxide (H_2_O_2_) availability, which may be limited in certain cancers. Hence, additional NPs have been developed to overcome this limitation. For example, PEGylated iron manganese silicate NPs (IMSNs) that are loaded with TGF-β inhibitor (TI), which are termed IMSN-PEG-TI, can induce macrophage polarization from M2 to M1 phenotype, potentially enhancing H_2_O_2_ production [89]. This nanozyme is noteworthy for having both CAT- and peroxidase (POD)-like properties. The CAT-like activity transforms H_2_O_2_ into O_2_ and the ensuing ^1^O_2_, while the POD-like activity generates hydroxyl radicals (•OH) or ROS, which in turn enhance the cytotoxicity of ^1^O_2_ against tumor cells [90]. In addition, NPs with dual phototherapy (PDT and photothermal therapy (PTT)) and dual catalytic abilities (CAT and POD activities) have been developed to improve the tumor inhibition rate up to 70.1% [91]. Moreover, MOF-derived NPs with dual phototherapy and dual catalytic ability, such as FePc/HNCSs (iron phthalocyanine/hollow nitrogen-doped carbon nanospheres), can further serve as contrast agents for ultrasound and magnetic resonance imaging (MRI), thereby guiding the therapy process and achieving an even better tumor inhibition rate to 96.3% [92].

In addition to the NPs stated above, hypoxia-specific nanotechnology has provided an alternative targeting strategy. In hypoxic TME, upregulation of transferrin receptor 1 (TfR1) in humans or T cell immunoglobulin mucin receptor 2 (TIM-2) in mice enhances the uptake of NP-loaded drugs by tumor cells [93]. This is facilitated by human ferritin nanocages (FTn) that target these receptors. Strategies combining the ability to target and relieve hypoxia have been recently developed. MnO_2_@Ftn, for instance, has been employed to transport MnO_2_ catalase-like nanozymes to the hypoxic locations precisely [94]. Acriflavine-loaded FTn has also been reported to specifically inhibit HIF-1α expression in hypoxic TME [93].

Hypoxic TME relieving could enhance the sensitivity of chemotherapies and immunotherapies, making the loading of tumor inhibitors onto these NPs a promising approach. For example, cisplatin-loaded FTn [93], or programmed death 1 (PD-1) blockade inhibitor-loaded multienzyme-mimic manganese oxide (MnOx) nanozyme [95] have been developed to combine chemo/immuno-therapeutic inhibitors and nanozymes for tumor therapy. Although NP loading may offer a novel way to improve EZH2i effectiveness in solid tumors, clinical application still faces challenges [96]. Firstly, the use of nanotechnology in tumors, particularly solid tumors, hinges on the enhanced permeability and retention (EPR) effect [97]. This effect allows NPs to infiltrate tumor tissue via leaky vasculature due to an ineffective lymphatic system that cannot efficiently drain them out. However, tumor heterogeneity results in accumulation of NPs, making it challenging to standardize the dosage. An insufficient dosage may fail to achieve the expected therapeutic effect, while an excessive dosage could exacerbate off-target effects and compromise long-term safety. For instance, one of the off-target effects, caused by TfR1 [93], which is also expressed in red cells and activated immune cells, can lead to undesired effects and toxicity [98]. Moreover, the bioavailability and biocompatibility of NPs are evaluated during their early development stages, and these factors may vary in clinical application due to individual differences and concurrent drug use [99]. Thus, ongoing scrutiny and assessment are crucial and urgent issues.

## Discussion and conclusions

In summary, the interplay between EZH2 and hypoxia/HIF-1α is a critical determinant of solid tumor progression and prognosis. This interaction is context-dependent and can be either enhanced or reduced by a variety of conditions, metabolites, and pathways. The relationship between EZH2 and HIF-1a impacts susceptibility and resistance to other cancer therapies such as AR antagonists and patient outcomes. Thus, novel strategies to target both EZH2 and hypoxia/HIF-1α may be useful to overcome tumor heterogeneity and aggressiveness.

However, for clinical application, it is crucial to investigate the regulatory network of EZH2 and HIF-1α from a more systematic perspective, such as angiogenesis and the immune landscape. While immunology has been researched [36–38], angiogenesis studies are scarce. Somewhat tangentially, HIF has been found to increase demethylation by boosting *KDM 4B/6B* transcription, leading to a transition of EZH2-mediated H3K27me3 to H3K27me2/1 at the *VEGF* promoter [100]. This reduction of methylation level enhances the transcription of *VEGF*, thereby promoting angiogenesis. Notably, this study did not involve co-culture or other experiments related to tumor cells, so the applicability of this regulatory pathway to TME remains to be explored.

A deeper understanding of the biology of the interplay between EZH2 and hypoxia will enable the precise stratification of solid tumors and guide the clinical application of EZH2is in the future. Also, several challenges need to be considered, including the ideal timing and manner of administration, possible side effects, and toxicity.
